# When Rivals Are Absent: Male Aggression Towards Females in Bluefin Killifish

**DOI:** 10.1002/ece3.71048

**Published:** 2025-03-13

**Authors:** Ratna Karatgi, Rebecca C. Fuller

**Affiliations:** ^1^ Program in Ecology Evolution, and Conservation Urbana Illinois USA; ^2^ Department of Evolution, Ecology, and Behavior School of Integrative Biology Urbana Illinois USA

**Keywords:** aggression, killifish, sex ratio, sexual conflict, spillover, territoriality

## Abstract

The process of obtaining mates, mating, and (potentially) caring for offspring is costly. While there are inherent costs to reproduction, behavioral interactions among individuals are often the primary drivers of reproductive costs. In many species, males frequently compete for territories and females; females may compete for food or males; males often harass females. Here, we sought to determine whether reproductive costs were primarily due to male/male competition, female/female competition, or male/female interactions in the bluefin killifish ( *Lucania goodei* ) using an unbiased approach that examined aggressive behaviors and interactions in both sexes. In this species, males hold small spawning territories during the breeding season, which they guard from competitors. Females visit the territories daily to spawn. To manipulate the potential for male and female competition and male/female interactions, we altered the sex ratio and density of each sex across four treatments (1 male: 1 female, 1 male: 3 females, 3 males: 1 female, 3 males: 3 females). Female mortality was higher than male mortality. Surprisingly, female mortality and male aggressive behaviors towards females (i.e., chases) were highest in treatments with a single male. Male–male aggression was present, but males often resolved these disputes via signaling by flaring their fins. There was little evidence for overt aggression among females. When males lack rivals, they turn their territorial defense towards females. These costs help explain why, in nature, females promptly leave male territories following spawning and join loose shoals with conspecific females and minnows.

## Graphical abstract

We examine male and female intrasexual aggression and male aggression towards females under different sex ratios and densities of each sex in bluefin killifish. Our findings show that females experience high costs of male aggression in the absence of competitor males, as indicated by increased female mortality and higher rates of male agonistic behavior when females are housed with a single male.

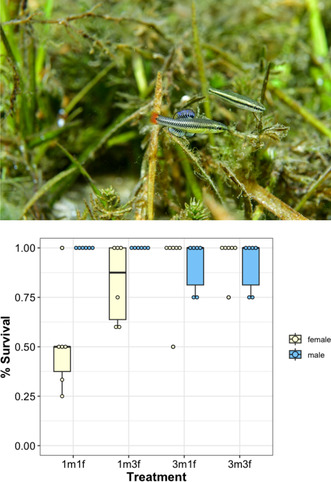

## 1 Introduction

Reproduction is costly in nearly all taxa. Females produce eggs, and males produce sperm, both of which involve a substantial investment of resources. Yet, many of the costs of reproduction stem from behavioral interactions, including aggression (Clutton‐Brock and Parker 1995; Chapman et al. 2003; Wedell et al. 2006; Pizzari and Bonduriansky 2010). Males compete with one another over access to mating territories or opportunities with females (Emlen and Oring 1977). Although not as well documented as male competition, females compete for access to males in many systems (Clutton‐Brock 2007; Rosvall 2011; Stockley and Campbell 2013; Hare and Simmons 2019; Schlupp 2021). Sexual conflict between males and females often results in costs for one or both sexes (Arnqvist and Rowe 1995; Pizzari and Bonduriansky 2010; Makowicz and Schlupp 2013). Teasing apart the effects of agonistic behaviors during reproduction can be complex, but it is necessary to understand the selective forces acting on males and females and the resulting mating system (Emlen and Oring 1977; Kvarnemo and Ahnesjö 1996; Wedell et al. 2006).

Manipulations of the operational sex ratio are often informative to understand the nature of sexual selection emanating from male competition, female competition, and male/female interactions (de Jong et al. 2012). The operational sex ratio (OSR) is often defined as the ratio of males ready to mate to females ready to mate at a given time (Emlen and Oring 1977). Other studies, such as (Clutton‐Brock and Parker 1992), define OSR based on the ratio of time individuals spend ready to mate, while (Kvarnemo and Ahnesjö 1996) use the proportion of males ready to mate relative to the total number of males and females ready to mate. Male‐biased sex ratios (more males ready to mate than females) are thought to promote competition among males over females. Indeed, several studies have found that as the number of reproductively available females decreases, the intensity of male intrasexual competition over mating opportunities increases (Schwagmeyer and Brown 1983; Colwell and Oring 1988; Kodric‐Brown 1988; Tejedo 1988; Kvarnemo et al. 1995; Cureton et al. 2010; Wacker et al. 2013; Chuard et al. 2016). Variations in environmental conditions, such as ambient temperature and spawning substrate quality, which make the operational sex ratio more male‐biased, also lead to increased male intrasexual competition (Zamudio et al. 1995; Forsgren et al. 1996, 2004; Kvarnemo 1996). In some species, the density of conspecifics, rather than the operational sex ratio, has a significant effect on male competition and aggression (Jirotkul 1999; Paciorek et al. 2020).

In recent years, competition among females has received more empirical support (Clutton‐Brock 2007, 2009; Rosvall 2011; Schlupp 2021). Female competition was historically associated with species such as pipefish where males choose mates, and females court males and compete for them (Colwell and Oring 1988; Berglund 1994; Ahnesjo 1995). However, recent evidence reveals significant competition among females, even in species where females are typically the choosers (Rosvall 2011; Schlupp 2021; Ranade et al. 2024).

Furthermore, reproductive costs often emerge due to behavioral interactions between males and females. Differences between males and females in the optimal mating rates result in the sexes having different optima for various traits, leading to sexual conflict (Trivers 1972; Rice 1996; Chapman et al. 2003). Male mate monopolization not only leads to intense competition between males but can also result in female harm by males. In species with internal fertilization, this can manifest as physical harm to female reproductive tracts, as seen in birds and many insects (Waage 1979; Arnqvist and Rowe 1995; Crudgington and Siva‐Jothy 2000), or through proteins present in seminal fluid that can reduce female lifespan, as seen in Drosophila (Chapman et al. 1995; Kuijper et al. 2006; Chapman 2018). An indirect effect of male harassment is that females spend more time avoiding male‐dominated regions, which can lead to a reduction in time spent foraging (Arnqvist 1989; Krupa and Sih 1993; Rowe 1994; Schlupp et al. 2001; Pilastro et al. 2003; Plath et al. 2003). As a result, females often pay a high cost due to male coercion, which can be seen in the form of a reduction in fecundity (McLain and Pratt 1999; Rossi et al. 2010), body condition (Watson et al. 1998; Makowicz and Schlupp 2013), and, in extreme situations, mortality (McEntee et al. 2023). Even in lek mating species where males cannot control access to females, male harassment of females is seen and can have dire consequences for females (Reale et al. 1996). Male mate harm is typically most intense during the periods of the breeding season when the sex ratio is highly male‐biased (Reale et al. 1996; Holand et al. 2006). Although male mate harassment has been documented in a broad range of taxa, much of this research is on internal fertilizers. Male mate harm in external fertilizers, including many fish and amphibians, has received less attention. In these species, male harassment of females is more likely to be through direct physical aggression rather than through mechanisms during copulation or post‐copulatory seminal fluids.

**FIGURE 1:**
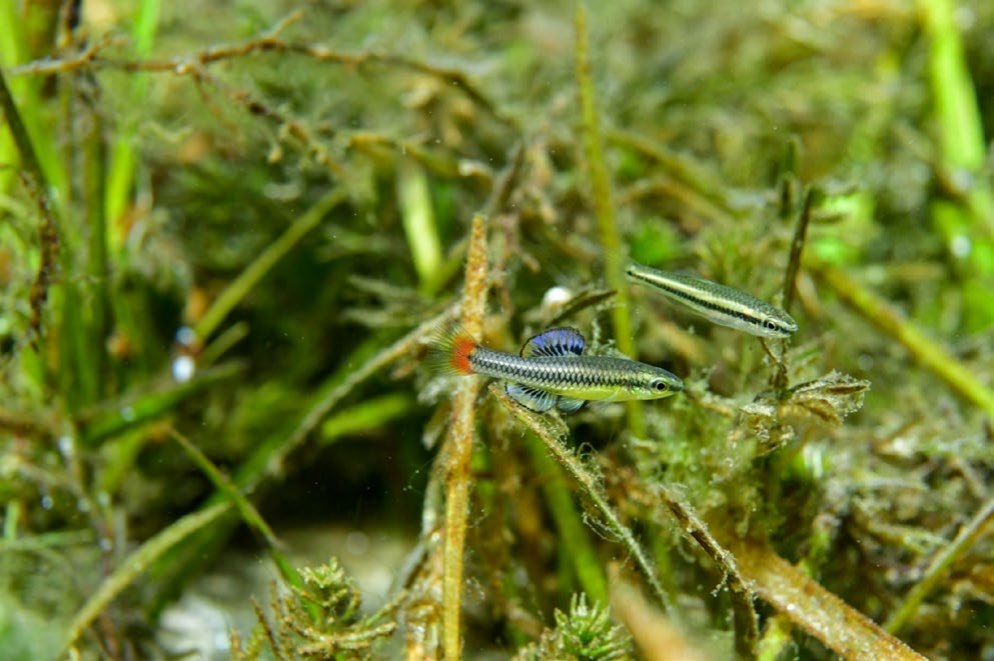
A male (left) and female (right) bluefin killifish ( *Lucania goodei* ) in a spring habitat. Photo credit—Thomas Waltzek.

In this study, we manipulate the operational sex ratio to examine the contributions of pre‐copulatory male–male, female–female, and male–female interactions on aggression and mortality in the bluefin killifish, *Lucania goodei* , a freshwater fundulid fish (Figure 1). In this species, males defend small territories during the reproductive season by flaring their fins, chasing, and attacking neighboring males, as well as occasionally directing aggression towards females and heterospecific fish (Foster 1967; Arndt 1971; Fuller 2001). Males quickly establish a dominance hierarchy that remains stable for a minimum of 6 weeks (McGhee and Travis 2010). When a female enters a male's territory, the male either courts the female or chases her away (Fuller 2001). During courtship, males swim in circles around the female, flash their fins, and flick their heads in the vicinity of the female (Foster 1967; Arndt 1971; Breder and Rosen 1966). If the female accepts the male, they proceed to pair spawn on aquatic vegetation (Fuller 2001); however, in laboratory settings, the most active males spawned with females, overriding female choice (McGhee et al. 2007). Following spawning, the female leaves the territory and joins loose shoals containing conspecific females, non‐territory holding males, and heterospecific fish (i.e., minnows). Although males guard their territories and are often aggressive well after spawning, there is no evidence that they provide parental care beyond territorial defense (Fuller and Travis 2001). In bluefin killifish, the spawning season is quite long, extending from February through September (Fuller 2001), with some authors reporting fish that breed year‐round (Arndt 1971). Females are highly iteroparous (Arndt 1971; Breder and Rosen 1966). Females become gravid multiple times each breeding season and spawn daily for about 2 weeks when gravid. Females have been observed to feed on eggs within a male territory (Fuller and Travis 2001).

Our specific goals were to determine whether the number of females, the number of males, and the sex ratio influence sex‐specific aggression and survivorship. We ask the following questions: (a) Does male intrasexual aggression vary between male‐biased and even sex ratio treatments? (b) Do females show intrasexual aggression? If so, does it differ between female‐biased and even sex ratio treatments? (c) Does male aggression towards females vary as a function of the density of each sex and the sex ratio? (d) Does male and female survivorship vary as a function of the density of each sex and the sex ratio?

## 2 Methods

This experiment aimed to assay the frequency of aggression in male and female bluefin killifish (both within and between the sexes) and to determine the extent to which aggression led to detrimental effects on survival. We manipulated the number of males and females of bluefin killifish in 110‐l tanks and observed behavior and survival.

Male and female bluefin killifish ( *Lucania goodei* ) were caught using seine nets from the Wakulla River, Florida. Fish were placed in aerated 18‐l buckets (with a maximum of 20 fish per bucket) and transported back to the University of Illinois. They were subsequently placed in a greenhouse in two 400‐l stock tanks (roughly hundred fish per tank at even sex ratios) with large sponge filters that aerated the water and removed nitrogenous wastes. Fish were moved to 110‐l tanks for the duration of the experiment where the tank water was continuously filtered and recirculated using hang‐on‐back filters. Fish were fed frozen brine shrimp until satiety daily. The tanks were furnished with green acrylic yarn mops, designed to mimic the natural aquatic vegetation these fish inhabit. These mops served as spawning sites, male territories, and as shelter for all the fish. The work was approved by the University of Illinois Institutional Animal Care and Use Committee (17184).

We manipulated the numbers of males and females to create treatments where competition and aggression were likely to differ between males and females. We created a treatment with a male‐biased sex ratio (3 males: 1 female) and a female‐biased sex ratio (1 male: 3 females). We also had two controls with an even sex ratio to control for the density of each sex (1 male: 1 female and 3 males: 3 females). Six replicates were performed across the four treatments (24 tanks in total). The tanks were large enough for all males to establish territories. The experiment was carried out between February 2020 and July 2020.

We conducted behavioral observations from June 2020 to July 2020. Fish were marked using a fluorescent elastomer dye injected underneath their skin, enabling individual identification within a tank (Northwest Marine Technology Inc.). Each fish in each tank was observed once for a duration of 5 min. During these observations, we recorded the number of chases and attacks exhibited. Aggressive behaviors occurred both before and after copulation. The number of fin flares and courtship bouts was also counted for males. For chases and attacks, we recorded the sex of the recipient of aggression by the focal individual. Behavioral observations were recorded using voice recordings, and the resultant audio files were transcribed using BORIS (Friard and Gamba 2016).

The experimental tanks were initially set up in late February 2020, and the fish remained in those conditions for 6 months. This extended duration was due to restricted lab access caused by the human COVID‐19 pandemic. During this time, tanks were censused, fish mortality was noted, and dead fish were replaced. We measured survival as the proportion of individuals alive in each treatment, which was the total number of individuals of each sex alive divided by the total number placed in the tank. In two instances (both in the 1m1f treatment) the male was replaced after high female mortality (two females dead in the span of 2 months) was observed in those tanks, presumable due to very high male aggression.

### 2.1 Statistical Analysis

#### 2.1.1 Aggression and Signaling

This experiment examined aggression within each of the sexes. Female aggression was rare and observed only in one instance. Therefore, we analyzed only male aggression and signaling behaviors. We compared two measures of male–male aggression (attacks and chases) between the two treatments where the number of males was three (3m1f and 3m3f). For both behaviors, we calculated the mean across the three males for each tank. Attacks were log‐transformed to meet the assumptions of analysis of variance (normal residuals, no heteroscedasticity). We performed a linear model in which we compared the levels of aggression between the two treatments (3m1f and 3m3f).

Male signaling was measured as the number of fin flares given by a male. Males flared their fins towards both males and females. We measured the mean number of fin flares performed for each tank. We log‐transformed the mean number of fin flares given to meet the assumptions of the analysis of variance. We performed a two‐way ANOVA, which examined the differences between the four treatments by looking at the effects of the number of males, number of females, and their interaction. Post hoc comparisons were made using Tukey's HSD from the emmeans package.

Male aggression towards females was in the form of chases and attacks. We analyzed the effects of the different treatments on both the total mean aggression experienced by females per treatment and the mean aggression experienced per female. The total mean aggression refers to the average number of aggressive behaviors observed in each treatment. In contrast, the mean per capita aggression accounts for the number of females present by dividing the total number of aggressive behaviors towards females by the number of females in each treatment, providing a measure of aggression experienced on an individual basis. Using a generalized linear model, we analyzed the effects of female density (1 or 3), male density (1 or 3), and their interaction on mean male chases and attacks towards females. Since the analysis was on non‐integer response variables that deviated significantly from normality and were highly over‐dispersed, we specified a gamma distribution for all four models of male aggression towards females. Post hoc comparisons were made using Tukey's HSD from the emmeans package.

In the one male three female (1m3f) treatment, we examined whether the single male distributed aggression evenly across all three females. We performed a generalized linear model to analyze the proportion of male aggression directed towards the most targeted female. The model included an intercept term to test whether the observed proportion differed from the expected proportion of 0.33. We specified a binomial distribution with a logit link function and included total aggression as weights. An offset term was applied to account for the expected proportion under equal aggression distribution. We checked for over‐dispersion but found none.

#### 2.1.2 Survival

We measured the survival of both males and females for each tank between February and July 2020. Animals died over the course of this experiment, and we replaced them. In two instances in the 1m1f treatment, the males were replaced due to high female mortality in those tanks. Since the replacement of males was not due to the death of the males, this replacement was not counted towards male survivorship. The survival of each sex was the total number of animals that lived at the end of the experiment divided by the total number of animals placed in that tank, including replacement animals. We first asked whether survival differed between males and females. We used a mixed model where we examined the effect of sex on survival, treating individual tanks as a random effect. We found a significant effect of sex and therefore analyzed the survival of each sex separately. We performed a generalized linear model for male and female survival, examining the effects of female density (1 or 3), male density (1 or 3), and their interaction. We specified a binomial distribution for all three models. We checked for overdispersion for each model but found none. All analyses of variance used type 3 comparisons and were conducted using the “car” package in R. The options for all analyses were set to options(contrasts = c (“contr.sum”, “contr.poly”)). All analyses were performed in R (version 4.4.0). Raw data and R codes are available at Dryad.

## 3 Results

### 3.1 Aggression

**FIGURE 2:**
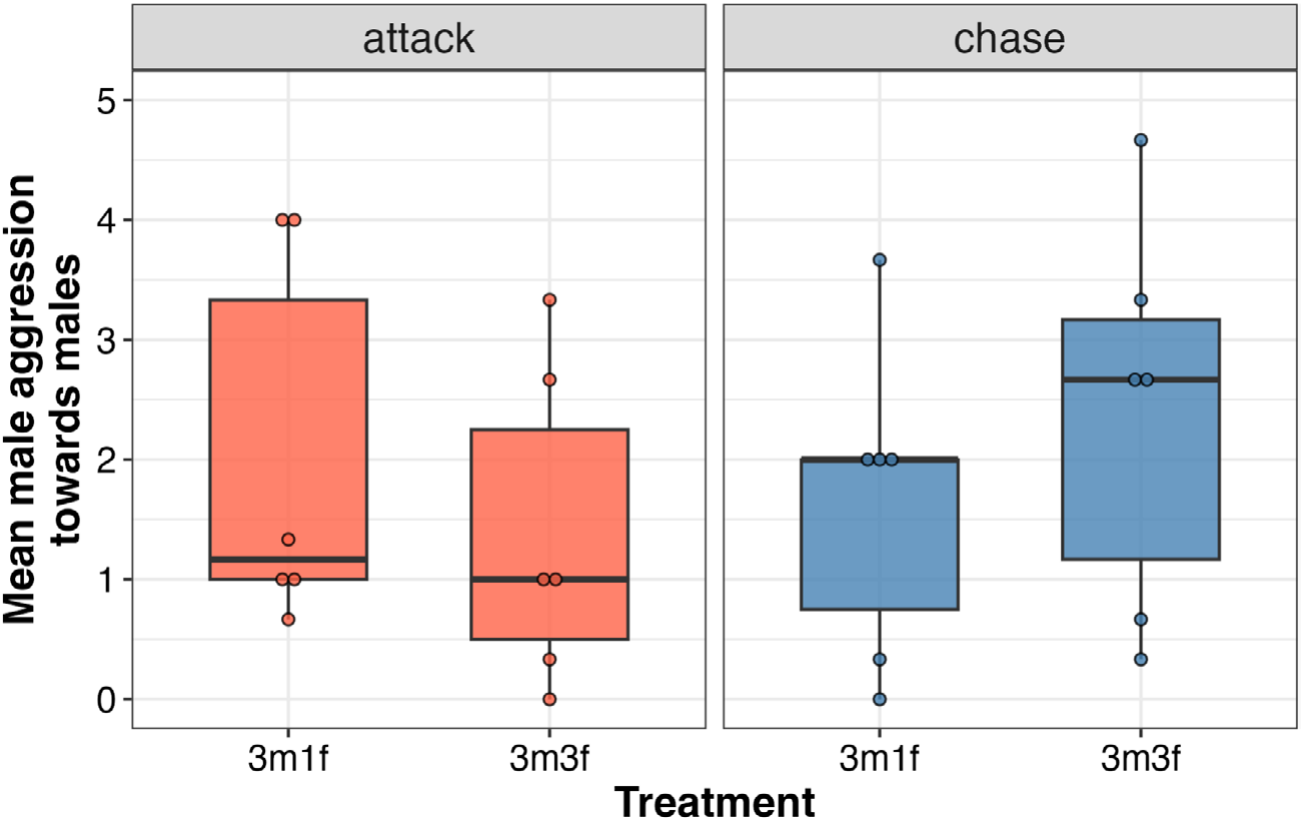
Box plots for the mean number of attacks and chases given by males. Each dot is the mean for one tank. The number of each sex is given on the *X*‐axis.

Females were rarely aggressive, and intrasexual female aggression was observed only in one instance (in the 3m3f treatment) across all the behavioral observations. Although males were aggressive towards each other when three males were present, male intrasexual aggression did not vary as a function of the number of females present, that is, between the 3m1f and 3m3f treatments (Figure 2, Table 1).

**TABLE 1:** Type 3 analysis of variance on the mean number of chases and attacks performed by males towards other males across the different sex‐ratio treatments (3m1f, 3m3f).

Terms	*F*	df (num, denom)	*p*
Mean chases			
Intercept	22.11	1,10	0.00
Number of females	0.70	1,10	0.42
b Mean attacks			
Intercept	32.32	1,10	0.00
Number of females	0.69	1,10	0.42

**FIGURE 3:**
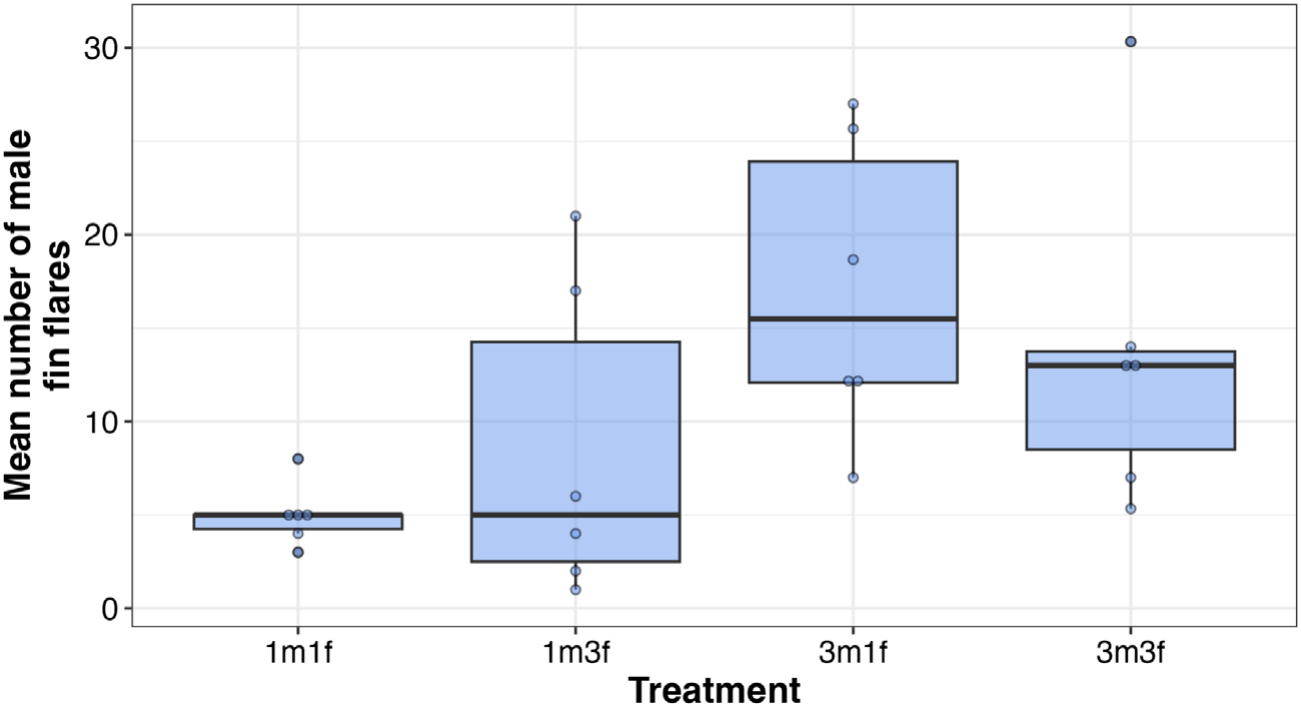
Box plots and mean numbers of fin flares by males across the four treatments. The number of each sex is given on the *X*‐axis.

Males signaled significantly more in the three male treatments (3m1f, 3m3f) compared to the one male treatments (1m1f, 1m3f). Male fin flares varied significantly due to the number of males (Figure 3, Table 2) but not the number of females or their interaction.

**TABLE 2:** Type 3 analysis of variance on the mean number of fin flares performed by males across all four treatments.

Terms	*F*	df (num, denom)	*p*
Intercept	317.96	1,20	0.00
Number of males	11.51	1,20	0.00
Number of females	0.06	1,20	0.81
Number of males × number of females	0.54	1,20	0.47

**FIGURE 4:**
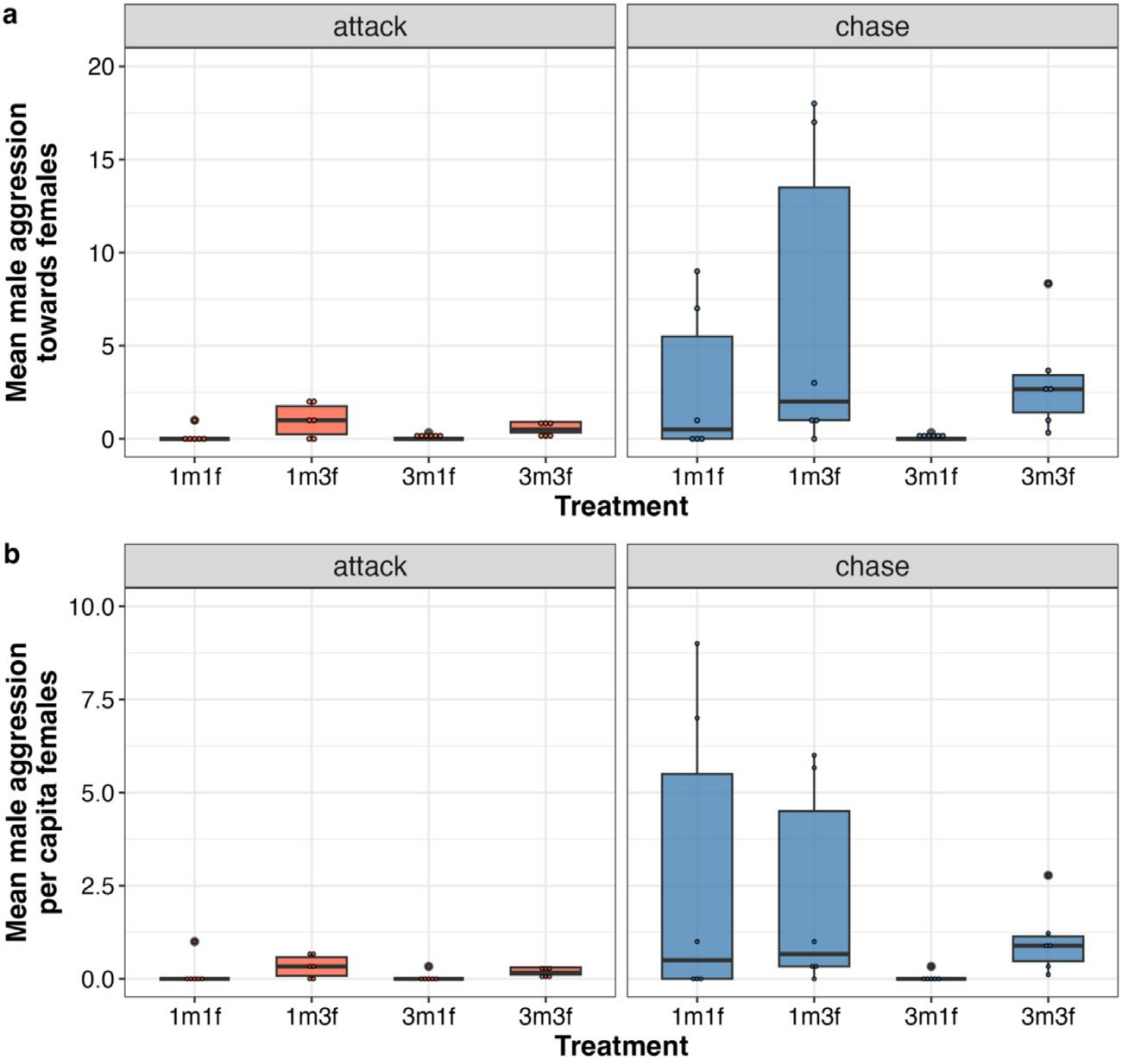
Box plots for the mean number of aggressive behaviors towards females by males across the four treatments. Each dot is an average for each tank. The number of each sex is given on the *X*‐axis. (a) Mean male aggression towards females. (b) Male aggression per capita female.

Male aggression towards females in the form of chases was significantly influenced by the number of males (Figure 4a, Table 3a) and the number of females, but not their interaction. Attacks towards females were much rarer than chases, and male attacks towards females were significantly influenced by the number of females (Table 3b), but not the number of males or the interaction of the two. Males showed more intersexual aggression when there were more females to be aggressive towards; that is, in the treatments with three females (1m3f, 3m3f) compared to one female (1m1f, 3m1f). In the 1m3f treatment, males did not distribute their aggression evenly among the females. Instead, they disproportionately targeted a single female, who frequently received around 60% of the total aggression (Table 4). In some replicates with a single female, we observed females hiding from males. Females experienced greater aggression from males in treatments with one male (1m1f, 1m3f) compared to three males (3m1f). More males did not lead to more aggression towards females.

**TABLE 3:** Type 3 analysis of deviance on the mean (a, b) and per capita (c, d) mean aggressive behaviors (chases and attacks) performed by males towards females across all four treatments.

Terms	*χ* ^*2*^	df	*P*
Mean chases			
Number of males	7.47	1	0.01
Number of females	8.55	1	0.00
Number of males × number of females	0.94	1	0.33
b Mean attacks			
Number of males	1.82	1	0.18
Number of females	12.57	1	0.00
Number of males × number of females	0.34	1	0.56
c Per capita mean chases			
Number of males	8.37	1	0.00
Number of females	0.66	1	0.42
Number of males × number of females	1.94	1	0.16
d Per capita mean attacks			
Number of males	1.41	1	0.23
Number of females	1.85	1	0.17
Number of males × number of females	0.01	1	0.92

**TABLE 4:** Summary of logistic regression analysis on the proportion of male aggression directed toward the most targeted female in the 1m3f treatment.

Terms	*Z*	df	*p*
Intercept	5.687	5	0.00

Male aggression per capita females in the form of chases was significantly influenced by the number of males (Figure 4b, Table 3c) but not the number of females or their interaction. Attacks towards females were much rarer than chases, and there were no patterns in male attacking behavior per capita females across the different treatments (Table 3d).

### 3.2 Survival

**FIGURE 5:**
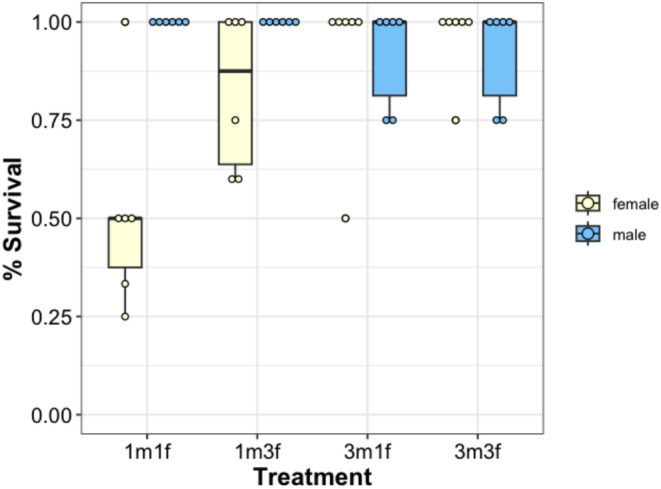
Box plots and raw data for survival for males and females across the four treatments. The number of each sex is given on the *X*‐axis. Survival was measured as the number of animals alive at the end of the experiment divided by the total number of animals put in the tank, including replacement animals.

Male survival was significantly higher than female survival (Figure 5, Table 5). Female survival was greatly reduced in treatments where there was one male in comparison to three males (Table 6a). Female survival was not significantly different between treatments with one or three females (Table 6a). The interaction between the number of males and the number of females did not account for a significant amount of variation in female survival (Table 6a). There were no effects of treatment on male survival (Table 6b).

**TABLE 5:** Type 3 analysis of deviance on the proportion of individuals alive as a function of sex.

Terms	*χ* ^2^	df	*p*
Sex	4.8057	1	0.03

**TABLE 6:** Type 3 analysis of deviance on the proportion of (a) females and (b) males alive across the four treatments.

Terms	*χ* ^2^	df	*p*
Female survival			
Number of males	6.31	1	0.01
Number of females	2.32	1	0.13
Number of males × number of females	0.08	1	0.78
b Male survival			
Number of males	2.20	1	0.14
Number of females	0.00	1	1.00
Number of males × number of females	0.00	1	1.00

## 4 Discussion

In this study, we examined how the density of each sex and sex ratio affect aggression, signaling, and survival in male and female bluefin killifish. We found high levels of female mortality when females were housed with a single male. Male aggression towards females was also higher in treatments with one male and multiple females, indicating that the density of males had the greatest influence on female survival and male intersexual aggression. There was aggression among males when multiple males were housed together, but the number of females (or the sex ratio) had little influence on these levels. Males also signaled via fin flares more when they were with other males, a finding that is in keeping with past work showing that fin flares precede aggression (Arndt 1971; Fuller 2001; McGhee et al. 2007). Below, we discuss the implications of these results.

Previous studies have shown that the male harassment of females tends to decrease as the number of females increases and the sex ratio becomes less male‐biased (Krupa and Sih 1993; Vepsäläinen and Savolainent 1995; Doutrelant et al. 2001; Cureton et al. 2010). However, we found unexpectedly high female mortality when females were housed with a single male, especially in the one male, one female treatment. In two cases, both in the one male, one female treatment, extreme male aggression led to multiple female deaths soon after their introduction to the tank, necessitating male replacements to allow behavioral observations. Since this study aimed to examine both male–female interactions and survival, this intervention was necessary. Despite this, females in the one male, one female treatment experienced significantly lower survivorship, and it is likely that survivorship would have been even lower without this intervention. In the one male, three female treatments, males often directed their aggression towards specific individual females while ignoring others.

In this experiment, females were housed continuously with at least one territorial male. An argument can be made that this is artificial because, in nature, females promptly leave the spawning territories to shoal with other females and minnows. However, our results shed light on this behavior in the wild; we argue that females leave male territories precisely because further interactions with territorial males are highly costly. Studies on mosquitofish show that shoaling reduces male harassment and increases foraging efficiency of females (Pilastro et al. 2003), with females preferring to associate with others in the presence of harassing males (Dadda et al. 2005). They also preferred to associate with larger females who were more likely to be targeted by a courting male (Agrillo et al. 2006). Non‐receptive female Trinidadian guppies chose to associate with receptive over non‐receptive females in order to receive lower male agonistic behaviors (Brask et al. 2012). In nature, bluefin killifish male aggression towards other males is high and primarily directed towards neighboring males, while aggression towards females is lower, as they can leave male territories (Fuller 2001). Our results indicate that male aggression can influence female association patterns in the wild.

Due to restricted lab access, behavioral observations were conducted a few months after fish were introduced to the tanks. Aggression towards conspecifics is typically highest at the first encounter and declines over time (Arndt 1971). While male aggression may have been higher initially, our observations still revealed clear patterns influenced by male density, suggesting meaningful behavioral trends were captured despite the delay.

An outstanding question is why males exhibit such excessive aggression, with several hypotheses still untested. We have observed females eating eggs or pecking at the spawning substrate, suggesting that male aggression could be a form of nest guarding to protect eggs from female predation. Although we did not explicitly test this, males were observed chasing or displacing females more frequently after they pecked at the spawning substrate compared to when they foraged elsewhere. While several species in the order *Cyprinodontiformes* display male parental care (Breder and Rosen 1966; Mank et al. 2005), studies on bluefin killifish found little evidence of increased egg survival due to male presence (Fuller and Travis 2001). However, these studies did not examine male behavior towards females or compare egg survival with and without females present.

A second hypothesis is that male aggression towards females represents the effects of the spillover of male intrasexual aggressive behavior. In nature, reproductive males spend much of their days defending their territories from neighboring males, and male aggression towards other males is tightly correlated with male mating success (Fuller 2001). Laboratory studies also show that male aggression and activity are key predictors of spawning success, outweighing female choice (McGhee et al. 2007). Without rival males, aggression may be misdirected towards females. In our lab, we had higher female survival in tanks with a single male–female pair when the males could see other males in neighboring tanks. Under these conditions, males tended to direct much of their attention towards their neighbors (Fuller, pers. obs).

Male aggression towards other males was lower than towards females, contrasting with patterns typically seen in the wild (Fuller 2001). While males did exhibit aggression towards each other, the frequency of aggression was unaffected by sex ratio, and they primarily involved signaling from a distance rather than direct physical conflict. Fin flaring—commonly associated with aggressive behavior in fish (Foster 1967)—was more frequently used during male–male interactions than male–female aggression, suggesting that males use signaling to assess rivals and avoid costly fights (Arndt 1971; Parker 1974; van Staaden et al. 2011). Similarly, females appear to mitigate the likelihood of aggression by distancing themselves from males and forming schools with other females (Arndt 1971).

In this study, females rarely exhibit aggression towards either sex. Although female intrasexual aggression is common in species where females typically exhibit greater mate choice—often driven by competition for mates or other resources (Clutton‐Brock 2009; Rosvall 2011; Ranade et al. 2024), our findings suggest it is less prominent in external fertilizers without significant parental care. Interestingly, similar patterns have been observed in sand gobies—another externally fertilizing species, but one with paternal care (Kvarnemo et al. 1995, 2024). It is possible that the smaller sample size in our study may have reduced our ability to detect female competition. However, the consistent absence of aggression across all replicate tanks suggests that it is genuinely infrequent among bluefin killifish females. A possible explanation is the species' prolonged breeding season, which could reduce competition by allowing females ample time with preferred males. Additionally, females often shoal with other conspecific and heterospecific fish when not in male territories (Arndt 1971), where aggressive behavior may not be advantageous or necessary.

In summary, our findings show that females incur significant reproductive costs due to male territorial behaviors, particularly when no competitor males are present. These costs are highlighted by the heightened aggression females face, which may contribute to reduced female survival in the presence of a single male. This pattern helps to explain observed female movement behaviors in the wild, where females typically avoid prolonged contact with territorial males. By highlighting the reproductive costs associated with mating in an iteroparous, external fertilizer species, our study enhances the understanding of the consequences of sexual conflict in species with external fertilization.

## Author Contributions

**Ratna Karatgi:** conceptualization (equal), formal analysis (lead), investigation (lead), methodology (equal), visualization (lead), writing – original draft (lead), writing – review and editing (lead). **Rebecca C. Fuller:** conceptualization (equal), formal analysis (supporting), methodology (equal), resources (lead), writing – original draft (supporting), writing – review and editing (supporting).

## Conflicts of Interest

The authors declare no conflicts of interest.

## Data Availability

All analyses were conducted in R version 4.4.4. The raw data and associated R‐code can be found at Dryad. https://doi.org/10.5061/dryad.wdbrv15z8.
